# Morphological characteristics of graft-host interface after ultra thin Descemet stripping automated endothelial keratoplasty (UT-DSAEK): impact of Descemetorhexis technique assessed by in vivo confocal microscopy (IVCM) and anterior segment optical coherence tomography (AS-OCT)

**DOI:** 10.1186/s12886-026-04823-8

**Published:** 2026-04-16

**Authors:** Alex Malandrini, Alberto Carnicci, Giulia Spadavecchia, Davide Marini, Giovanni Rubegni, Antonio Moramarco, Gian Marco Tosi

**Affiliations:** 1 Unit of Ophthalmology, Department of Medicine, Surgery and Neuroscience, University of Siena, Siena, Italy; 2 Ophthalmology Unit, Dipartimento di Scienze Mediche e Chirurgiche, Alma Mater Studiorum, University of Bologna, Bologna, Italy; 3 Ophthalmology Unit, Santa Maria delle Croci Hospital, Ravenna, Italy

**Keywords:** Ultra-thin Descemet stripping automated endothelial keratoplasty (UT-DSAEK), Graft–host interface, Descemetorhexis technique, Descemet stripping, In vivo confocal microscopy (IVCM), Anterior segment optical coherence tomography (AS-OCT), Interface reflectivity, Mean grey intensity (MGI), Fuchs’ endothelial corneal dystrophy (FECD), Endothelial keratoplasty

## Abstract

**Background:**

The graft–host interface plays a critical role in visual recovery after Ultra-Thin Descemet Stripping Automated Endothelial Keratoplasty (UT-DSAEK). This study evaluated graft–host interface morphology using quantitative Mean Grey Intensity (MGI) analysis, comparing three different Descemetorhexis techniques: ophthalmic viscosurgical device (OVD)-assisted, balanced salt solution (BSS)-assisted and air-assisted stripping.

**Materials and methods:**

This retrospective comparative study included 59 eyes with Fuchs’ endothelial corneal dystrophy (FECD) undergoing UT-DSAEK. According to Descemetorhexis technique, patients were divided into three groups: OVD-assisted (VISCO, *n* = 21), BSS-assisted (BSS, *n* = 20), and air-assisted (AIR, *n* = 18). The primary outcome was graft–host interface reflectivity, quantitatively assessed using MGI measurements obtained with ImageJ software on in vivo confocal microscopy (IVCM) and anterior segment optical coherence tomography (AS-OCT). Best-corrected visual acuity (BCVA) was evaluated as a secondary outcome. Examinations were performed at 6 and 12 months postoperatively. Longitudinal comparisons were conducted using repeated-measures analysis of variance.

**Results:**

BCVA significantly improved over time in all groups (*p* < 0.001). At 6 months, the AIR group demonstrated significantly better visual acuity recovery and the lowest interface reflectivity on both IVCM and AS-OCT (*p* < 0.01), whereas the VISCO group exhibited the highest values on both modalities (*p* < 0.001). The BSS group showed intermediate results. At 12 months, reflectivity significantly decreased in all groups on both imaging modalities (*p* < 0.001). Although the VISCO group showed the greatest absolute reduction over time, its reflectivity values remained slightly higher than those of the AIR and BSS groups. The AIR group maintained the lowest reflectivity at both time points.

**Conclusions:**

Descemetorhexis technique is significantly related to differences in early graft–host interface morphology following UT-DSAEK. OVD-assisted stripping is associated with higher early reflectivity, whereas BSS and particularly air-assisted techniques promote faster optical clearing and visual recovery. Over time, progressive interface remodelling leads to a reduction in reflectivity across all techniques, suggesting a tendency toward long-term convergence in optical quality regardless of the intraoperative medium used.

## Background

Descemet Stripping Automated Endothelial Keratoplasty (DSAEK) is a widely adopted surgical procedure to manage corneal endothelial diseases such as Fuchs’ endothelial corneal dystrophy (FECD) and pseudophakic bullous keratopathy. When compared to penetrating keratoplasty (PK), DSAEK offers several benefits, including faster visual rehabilitation, reduced postoperative astigmatism, and fewer wound complications [1–3]. 

Although DSAEK generally provides favorable outcomes, unsatisfactory visual results can still occur in some patients, even when the graft appears clear. To enhance visual outcomes without compromising safety, Ultra-Thin DSAEK (UT-DSAEK) — characterized by donor lenticules thinner than 100 μm — has gained increasing popularity. This technique provides visual results comparable to Descemet Membrane Endothelial Keratoplasty (DMEK), while maintaining the procedural simplicity and lower complication rates of conventional DSAEK [4–6].

Nevertheless, both DSAEK and UT-DSAEK remain susceptible to interface-related abnormalities, which are among the most relevant postoperative complications. In the literature, terms such as ‘Textural Interface Opacities’, ‘interface wave-like deposits’, ‘reticular haze’, and ‘ground glass interface haze’ are frequently used interchangeably to describe a specific type of interface haze marked by elevated light scatter, which is attributed to microscopic irregularities and the abnormal deposition of inert material at the graft-host junction [7–9].

However, this phenomenon represents just one manifestation within a broader spectrum of interface pathologies that may occur after endothelial keratoplasty. The pathogenesis of these alterations is multifactorial and includes factors such as surgical technique, donor tissue quality, residual interface fluid, particulate contamination, stromal irregularities, and inflammatory cellular infiltration [10, 11].

In the early postoperative period, incomplete graft adhesion or inadequate air tamponade may result in interface fluid or partial graft detachment, which can often be managed by intracameral air reinjection (‘rebubbling’) [12]. Other cases involve residual particulate matter — such as epithelial cells, viscoelastic remnants, or surgical debris [13] — entrapped between graft and host, which can produce localized opacity. Persistent detachment or chronic inflammation may subsequently lead to interface fibrosis, a more permanent form of opacity associated with long-term loss of transparency [14].

More severe but less frequent complications include interface infectious keratitis [15] and epithelial ingrowth [16] at the graft interface, both of which may require surgical intervention. Additional interface abnormalities, such as folds or wrinkling due to curvature mismatch, can also contribute to optical degradation [7, 17, 18].

Studies with in vivo confocal microscopy (IVCM) [19] and anterior segment optical coherence tomography (AS-OCT) [20] have revealed that increased interface reflectivity corresponds to worse best-corrected visual acuity (BCVA).

To date, the literature lacks a comprehensive morphological evaluation of the graft-host interface following DSAEK; most previous investigations have evaluated interface characteristics using either IVCM or AS-OCT individually, and standardized longitudinal analyses combining these two complementary imaging modalities remain limited. Moreover, many studies provide primarily qualitative descriptions of interface abnormalities rather than objective quantitative measurements.

In this context, a detailed evaluation of the graft–host interface remains clinically relevant to better understand the mechanisms influencing visual recovery after endothelial keratoplasty. The present study therefore primarily focuses on the structural characterization of the interface, while visual acuity was assessed to provide a functional context to the morphological findings.

In this study, IVCM and AS-OCT were integrated with a standardized quantitative assessment of interface reflectivity based on Mean Grey Intensity (MGI). This multimodal and quantitative approach allows a more comprehensive assessment of graft–host interface morphology over time.

In addition, although there have been significant progresses, comparative data on the effect of different Descemetorhexis techniques on interface reflectivity and visual outcomes are still limited. This study attempts to fill this gap by comparing the effects of different Descemet stripping techniques—ophthalmic viscosurgical device (OVD)-assisted, balanced salt solution (BSS)-assisted, and air-assisted— on the graft-host interface reflectivity patterns following UT-DSAEK, providing longitudinal morphological data.

## Materials and methods

### Study design and participants

This retrospective comparative study was conducted in accordance with the principles of the Declaration of Helsinki and received approval from the Hospital Ethics Committee (protocol number 29569, approved on September 15, 2025). Both verbal and written informed consent were obtained from all participants prior to enrollment.

This comparative, retrospective study included 59 eyes from 59 patients diagnosed with Fuchs’ endothelial corneal dystrophy (FECD). All the patients underwent UT-DSAEK at the Ophthalmology Unit of Le Scotte University Hospital (Siena, Italy) between January 2023 and September 2024. Patients were monitored through regular follow-up visits until September 2025, ensuring consistent postoperative evaluation and data collection. Given the retrospective nature of the study, the choice of Descemetorhexis technique (OVD-assisted, BSS-assisted, or air-assisted) was based on surgeon preference and intraoperative considerations rather than predefined allocation criteria.

Inclusion criteria included patients aged 50 to 80 years with a clinically documented diagnosis of FECD. Only pseudophakic eyes without significant posterior capsule opacification (PCO), or eyes that had already undergone Nd: YAG laser capsulotomy, were included.

Patients with non-FECD ocular conditions affecting the endothelium, a prior history of intraocular surgery other than phacoemulsification, or coexisting disorders affecting visual acuity — such as glaucoma or age-related macular degeneration — were excluded from the study.

Furthermore, patients with complicated UT-DSAEK procedures and DSAEK grafts > 100 μm were excluded.

### Surgical technique

All procedures were performed by a single experienced surgeon (A.M.) with peribulbar anesthesia. Donor corneal tissues with endothelial cell density ≥ 2500 cells/mm² were obtained from the Regional Tissue and Cell Bank of Tuscany (Lucca, Italy).

Donor tissue was punched using CORONET^®^ Donor Corneal Trephine Vacuum Punch (Network Medical Products Ltd, North Yorkshire UK), with a diameter between 7.5 mm and 8.0 mm, depending on recipient corneal diameter and surgeon preference.

**Fig. 1:**
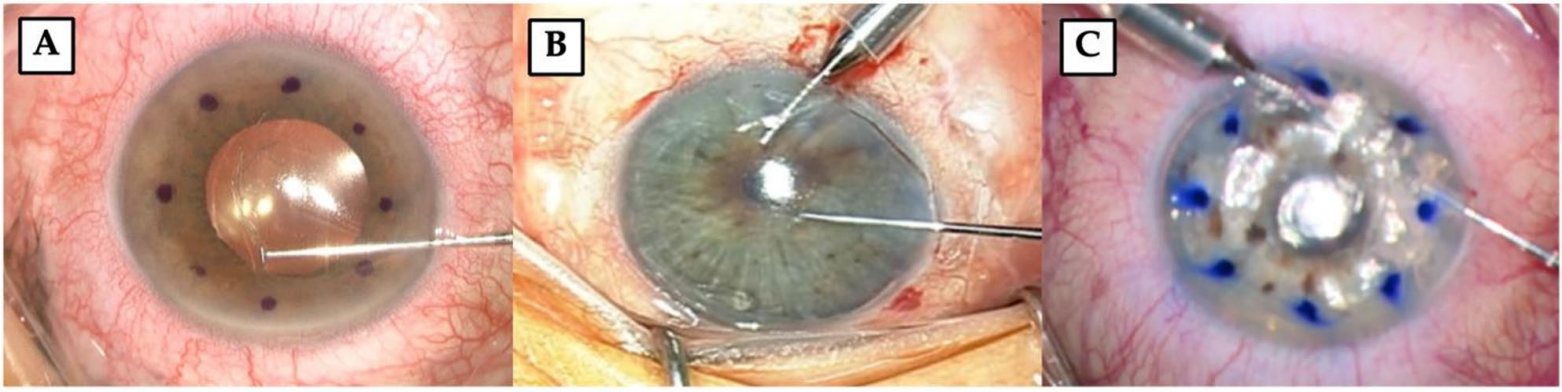
Illustration of the three Descemetorhexis techniques used in the study. (**A**) OVD-assisted technique. (**B**) BSS-assisted technique. (**C**) Air-assisted technique

The Descemetorhexis was performed using one of the following three techniques (Fig. 1):

- Ophthalmic viscosurgical device (OVD)-assisted (Fig. 1A): OVD (Proviscs^®^, Alcon Laboratories, Fort Worth, TX, USA) was injected into the anterior chamber.
- Balanced Salt Solution (BSS)-assisted (Fig. 1B): a 23-Gauge anterior chamber maintainer self-retaining (E. Janach srl, Como, Italy) was inserted into the anterior chamber through an infero-temporal incision and connected to an elevated BSS bottle.
- Air-assisted (Fig. 1C): the same anterior chamber was connected to a 3-way tap, with both the BSS infusion and the air line supplied by the Stellaris PC Vitreoretinal Surgery System fluid-air exchange pump (Bausch & Lomb, Rochester, NY, USA). Descemet stripping was then performed under continuous air flow [21].

Following descemetorhexis, the donor lenticule was inserted with Busin glide, unfolded, and positioned appropriately. An air bubble was used to tamponade the graft against the recipient stroma, and patients were instructed to remain in a supine position for a minimum of 3 h to support graft adhesion.

### Physical examination and interface analysis

All patients underwent routine postoperative follow-up visits during the first year after surgery. To analyze the evolution of interface, two representative timepoints were selected: an early postoperative evaluation at 6 months and a later evaluation at 12 months. These timepoints were chosen to assess both the acute healing response and the potential remodeling or normalization of the graft-host interface over time.

During these follow-ups, a complete ophthalmological evaluation was performed, including a measurement of best-corrected visual acuity (BCVA) expressed in logMAR units, providing a standardized measure of visual function. In addition, both AS-OCT and IVCM were performed to assess graft adherence and the structural characteristics of the graft–host interface in the three different Descemetorhexis techniques.

**Fig. 2:**
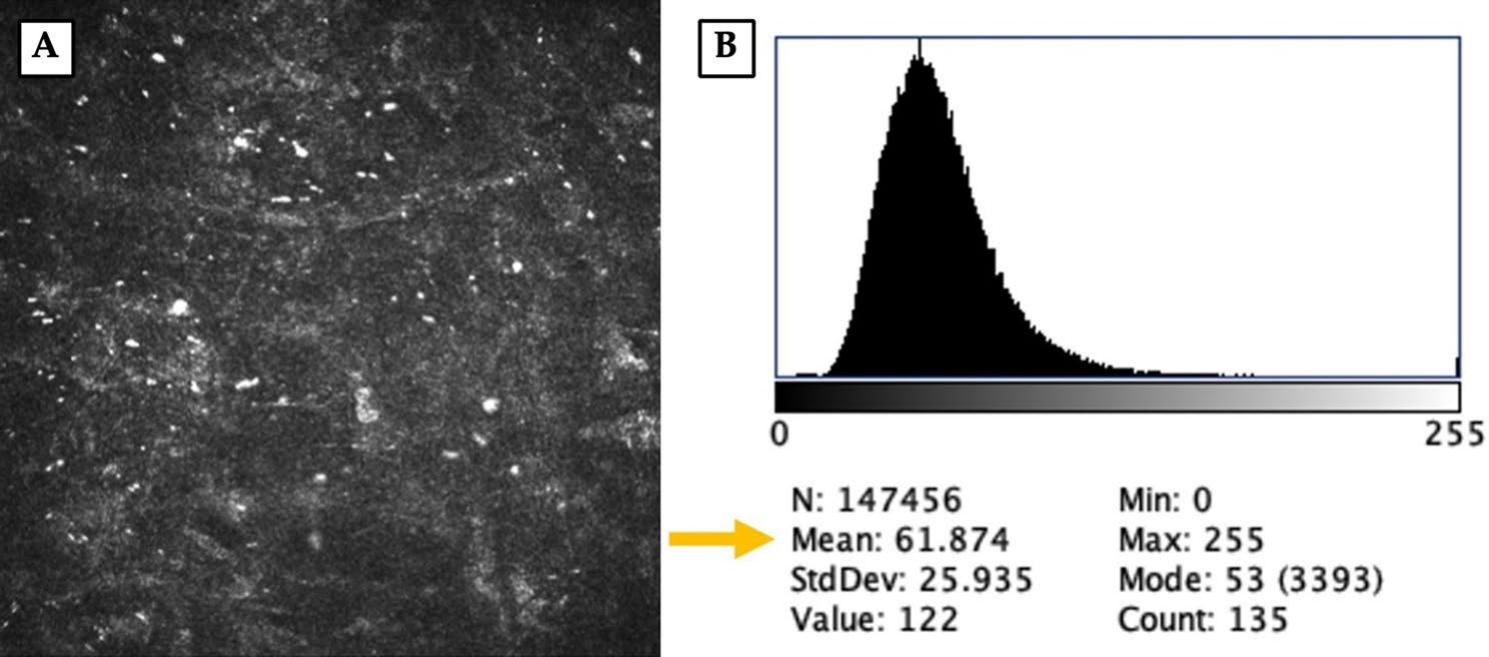
(**A**) Representative in vivo confocal microscopy (IVCM) image of the graft–host interface. (**B**) Optical density of the interface was calculated as the mean value of the grey intensity using the histogram function of the Image J software applied on the entire IVCM scan

**Fig. 3:**
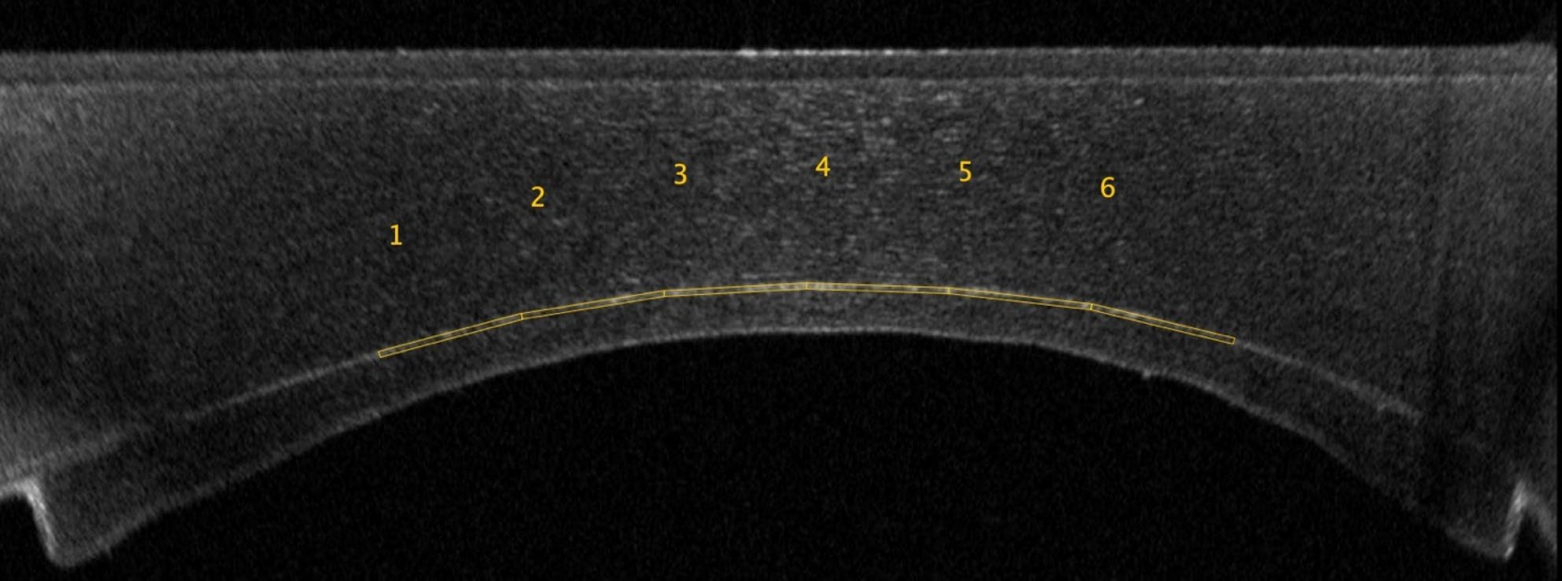
The central interface was split into six thin rectangles (6 × 150 pixels each) using the ImageJ software, and the reflectivity was determined as the mean grey intensity averaged among the six portions and across at least three AS-OCT scans

IVCM was performed using the Heidelberg Retina Tomograph II with Rostock Cornea Module (Heidelberg Engineering GmbH, Germany) by the same experienced examiner (A.C.) for all eyes. Before the exam, a drop of topical anesthetic was instilled; the patient was then asked to steadily fixate a target while the central cornea was scanned. Each examination took approximately 3 min, recording multiple images with a 1.5 mm distance between successive images along the z-axis. The host-donor interface area was identified as the acellular zone found proceeding from epithelium to endothelium (Fig. 2A); the presence of particulate debris within that zone further guides the delineation of the true interface plane. Additionally, pachymetric data obtained from AS-OCT were used to confirm the corresponding depth. The optical density of the interface was determined as the Mean Grey Intensity (MGI) using the histogram function of the image processing software ImageJ (Software z project tool, version 1.54 g, National Institute of Health, Bethesda, MD, USA), after averaging at least three IVCM scans (Fig. 3B).

AS-OCT was performed with MS-39 (Costruzione Strumenti Oftalmici, Florence, Italy) by the same experienced examiner (G.S.), minimizing inter-observer variability, and every effort was made to avoid the reflex saturation beam while maintaining central alignment as much as possible. For the quantitative analysis, the straightened corneal OCT views (‘applanated’ sections) provided by the MS-39 software were used. Images were acquired from the central cornea, and measurements were repeated three times per patient to increase accuracy. The reflectivity was determined as the MGI of the central interface split into six thin rectangles (6 × 150 pixels each) for practical purpose using the ImageJ software (Software z project tool, version 1.54 g, National Institute of Health, Bethesda, MD, USA) and then averaged across three AS-OCT scans (Fig. 3).

### Statistical analysis

Statistical analysis was performed using SPSS version 29 (IBM Corp, Armonk, NY, USA). The three groups were compared at baseline with one-way analysis of variance (ANOVA) for continuous variables and Chi-square test for categorical variables. The main effect of time, group and their interaction on each of the three main outcomes (BCVA, IVCM, and AS-OCT measurements) were assessed using a two-way repeated measure ANOVA. When significant effects were observed, post hoc pairwise comparisons with Bonferroni correction were performed to identify differences between specific groups at each follow-up. A p-value less than 0.05 was considered statistically significant.

## Results

### Baseline characteristics

59 eyes of 59 subjects with FECD were enrolled in the study and underwent UT-DSAEK. The cohort included 35 females and 24 males, with a mean age of 72.03 (range, 51–80) years. Demographic characteristics, key clinical parameters, and group distribution of the patient population enrolled are listed in Table 1. The three groups (VISCO, BSS, and AIR) were comparable in terms of age, gender distribution, laterality, and baseline BCVA, with no statistically significant differences among them (all *p* > 0.05). Similarly, the timing of postoperative evaluations (Visit 1 and Visit 2) was consistent across groups.

**Table 1:** Baseline demographic and clinical characteristics of patients in the three treatment groups: ophthalmic viscosurgical device-assisted (VISCO), balanced salt solution-assisted (BSS), and air-assisted (AIR). Data are presented as mean ± standard deviation or number (female/male; right/left)

	VISCO (*n* = 21)	BSS (*n* = 20)	AIR (*n* = 18)	*p*
Age at surgery (years)	72.6 ± 7.7	74.9 ± 5.9	68.6 ± 11.7	0.181
Gender (F/M)	12 / 9	13 / 7	10 / 8	0.813
Eye (R/L)	9 / 12	10 / 10	9 / 9	0.871
BCVA baseline (logMAR)	0.84 ± 0.22	0.98 ± 0.30	0.88 ± 0.33	0.274
Visit 1 (months)	5.50 ± 0.94	5.26 ± 0.81	5.09 ± 0.74	0.303
Visit 2 (months)	11.92 ± 1.99	12.38 ± 1.77	11.42 ± 1.77	0.287

### Best corrected visual acuity (BCVA)

**Fig. 4:**
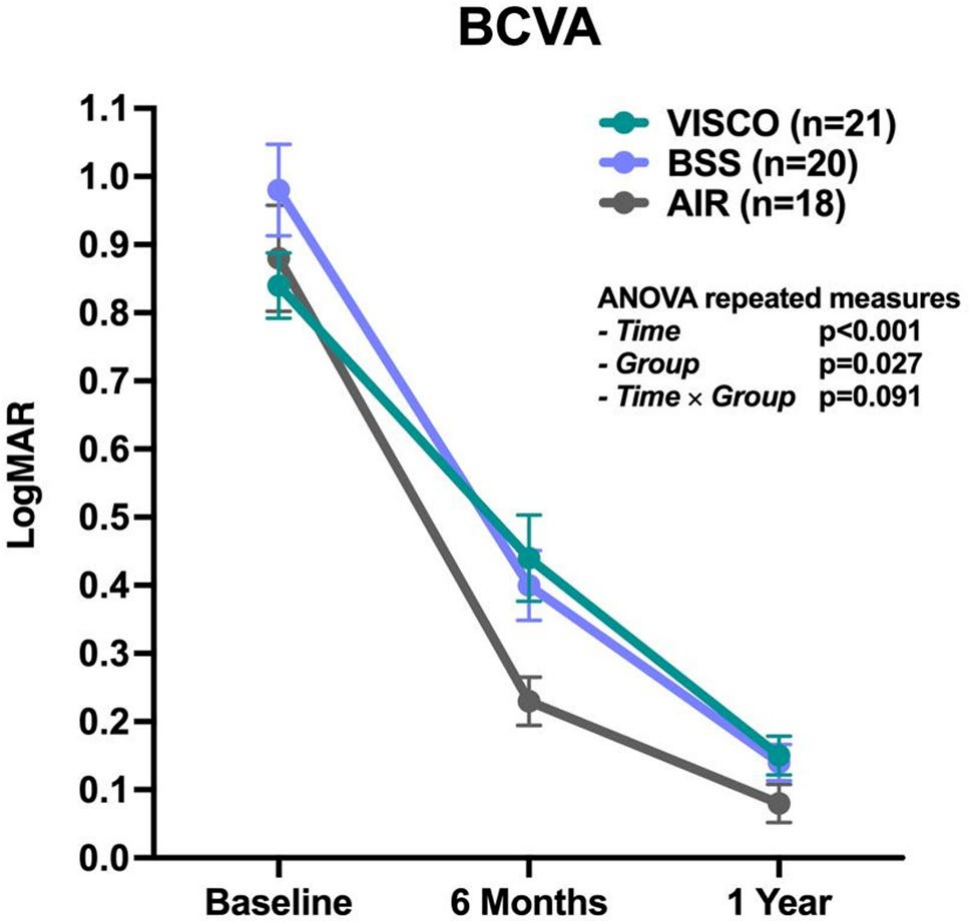
Longitudinal assessment of best-corrected visual acuity (BCVA) in the three Descemetorhexis technique groups at baseline, 6 months, and 12 months after UT-DSAEK. Values are expressed in logMAR. P-values corresponding to the effects of time, group, and time × group from the repeated-measures ANOVA are reported

Although the primary aim of this study was a morphological analysis of the corneal interface, BCVA was assessed at 6 and 12 months after UT-DSAEK to evaluate functional recovery across the three Descemetorhexis technique groups (Fig. 4). The mean BCVA values for each group are reported in Table 2, while the results of the repeated-measures ANOVA are presented in Table 3.

A significant improvement in BCVA was observed over time (*p* < 0.001), indicating a general visual recovery during the 12-month follow-up across all groups. Differences among the three surgical techniques were also statistically significant (*p* = 0.027), while no significant interaction between time and group was found (*p* = 0.091), suggesting that the rate of visual improvement was comparable between groups.

At 6 months, mean BCVA values were 0.44 ± 0.29 logMAR in the VISCO group, 0.40 ± 0.23 logMAR in the BSS group, and 0.23 ± 0.15 logMAR in the AIR group. Post hoc Bonferroni comparisons showed that the AIR group had significantly better BCVA than both the VISCO (*p* = 0.009) and BSS (*p* = 0.039) groups.

By 12 months, mean BCVA improved to 0.15 ± 0.13 logMAR in the VISCO group, 0.14 ± 0.12 logMAR in the BSS group, and 0.08 ± 0.12 logMAR in the AIR group. At this time point, visual outcomes were comparable across all groups, with no statistically significant differences observed.

### In vivo confocal microscopy (IVCM)

**Fig. 5:**
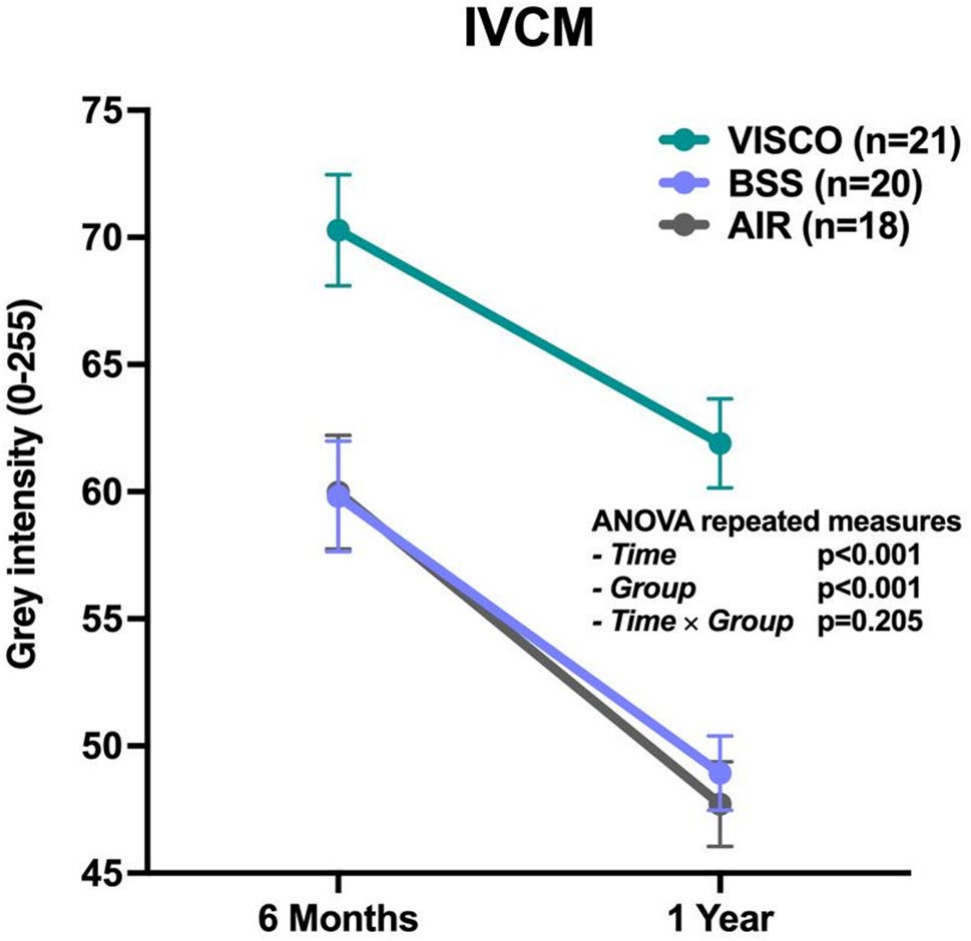
Quantitative analysis of interface measured by In Vivo Confocal Microscopy (IVCM) at 6 and 12 months after UT-DSAEK. Reflectivity was calculated including interface debris and values are expressed as Mean Grey Intensity (MGI) of the graft–host interface. P-values corresponding to the effects of time, group, and time × group from the repeated-measures ANOVA are reported

**Fig. 6:**
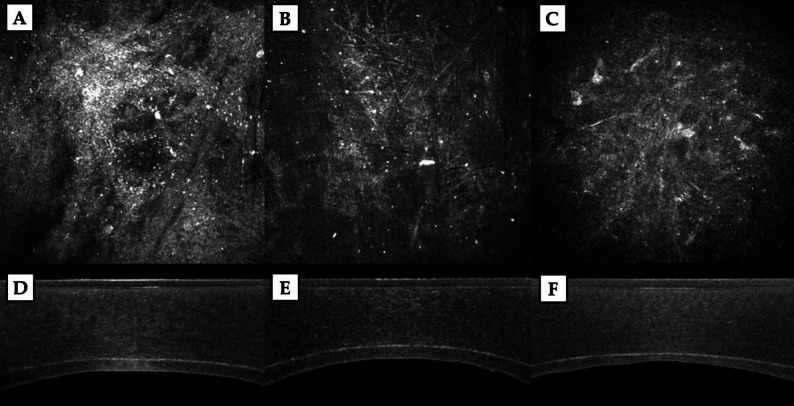
Interface evaluation by in vivo confocal microscopy (IVCM, **A**–**C**) and anterior segment optical coherence tomography (AS-OCT, **D**–**F**) across the different Descemetorhexis groups. (**A**, **D**) OVD-assisted technique; (**B**, **E**) BSS-assisted technique; (**C**, **F**) Air-assisted technique

Interface reflectivity analysis based on IVCM images was performed at 6 and 12 months after UT-DSAEK to evaluate the morphological characteristics of the graft–host interface (Fig. 5). Reflectivity was assessed as MGI of the interface, calculated including the contribution of interface debris (Fig. 6). The MGI values for each group are reported in Table 2, while the results of the repeated-measures ANOVA are shown in Table 3.

A significant reduction in interface reflectivity over time was observed across all groups (*p* < 0.001). Significant differences were also found among the three surgical techniques (*p* < 0.001), whereas no significant interaction between time and group was detected (*p* = 0.205), indicating a similar trend of reduction across groups.

At 6 months, post hoc Bonferroni comparisons showed that the VISCO group exhibited significantly higher MGI values (70.28 ± 10.01) compared to both the BSS (59.82 ± 9.73, *p* = 0.001) and AIR (59.98 ± 9.47, *p* = 0.002) groups.

Similarly, at 12 months, MGI remained significantly higher in the VISCO group (61.90 ± 8.03) compared to the BSS (48.93 ± 6.51, *p* = 0.001) and AIR (47.71 ± 7.08, *p* = 0.002) groups.

### Anterior segment – optical coherence tomography (AS-OCT)

**Fig. 7:**
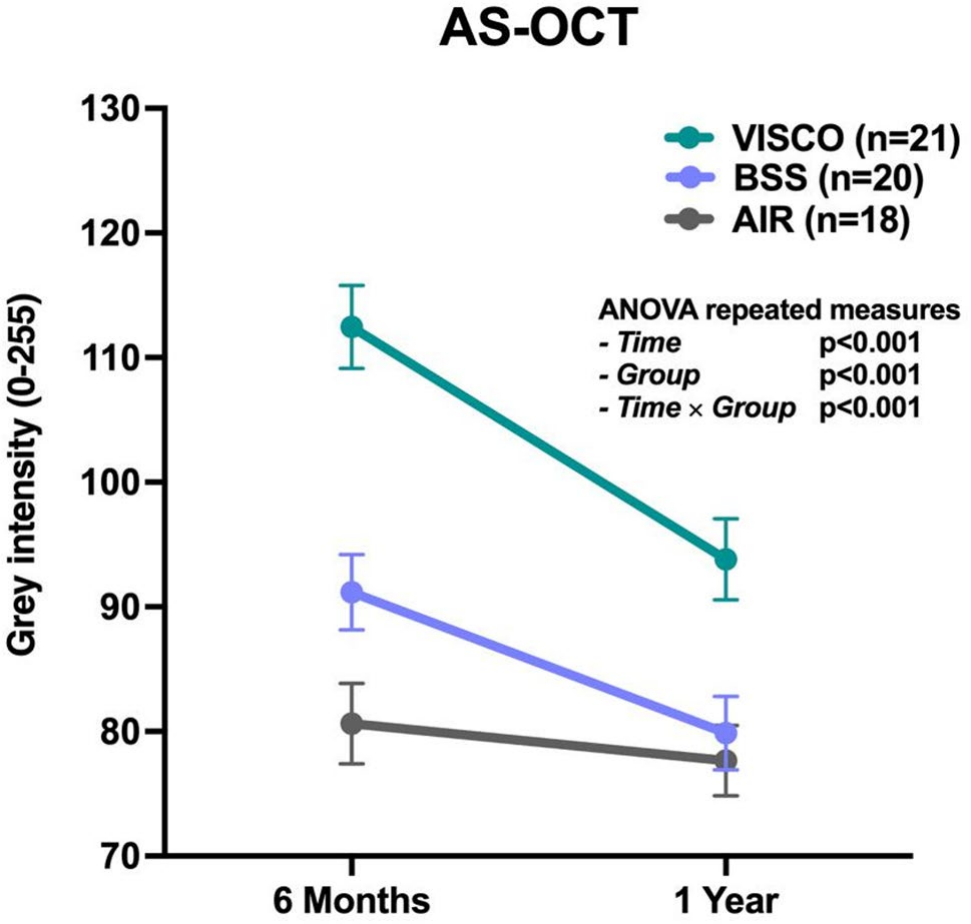
Quantitative assessment of interface reflectivity by Anterior Segment Optical Coherence Tomography (AS-OCT) at 6 and 12 months after UT-DSAEK. Reflectivity values are expressed as Mean Grey Intensity (MGI) of the graft–host interface. P-values corresponding to the effects of time, group, and time × group from the repeated-measures ANOVA are reported

Interface reflectivity analysis based on AS-OCT images was performed at 6 and 12 months after UT-DSAEK to assess structural characteristics of the graft–host interface (Fig. 7). Reflectivity was expressed as MGI within the interface region (Fig. 6). The MGI values for each group are described in Table 2 and the results of the repeated-measures ANOVA are presented in Table 3.

Overall, significant differences in interface reflectivity were observed among the three surgical groups (*p* < 0.001) and over time (*p* < 0.001). Moreover, a significant interaction between time and group (*p* < 0.001) showed that the extent of reflectivity decreases varied among the different surgical techniques.

At 6 months, mean MGI values were 112.45 ± 15.26 in the VISCO group, 91.17 ± 13.49 in the BSS group, and 80.64 ± 13.70 in the AIR group. Post hoc Bonferroni comparisons showed that VISCO exhibited significantly higher reflectivity than BSS (*p* < 0.001) and AIR (*p* < 0.001), and that BSS reflectivity was significantly greater than AIR (*p* = 0.026).

At 12 months, interface reflectivity decreased in all groups, reaching 93.82 ± 14.90 in the VISCO group, 79.86 ± 13.19 in the BSS group, and 77.66 ± 11.95 in the AIR group. Bonferroni post hoc analysis confirmed that the VISCO group maintained significantly higher reflectivity compared to both BSS (*p* = 0.002) and AIR (*p* < 0.001).

**Table 2:** Functional and morphological outcomes at 6 months and 12 months after UT-DSAEK in the three Descemetorhexis technique groups. Values are presented as mean ± standard deviation

		VISCO (*n* = 21)	BSS (*n* = 20)	AIR (*n* = 18)
BCVA (logMAR)	6 months	0.44 ± 0.29	0.40 ± 0.23	0.23 ± 0.15
	1 year	0.15 ± 0.13	0.14 ± 0.12	0.08 ± 0.12
IVCM (MGI)	6 months	70.28 ± 10.01	59.82 ± 9.73	59.98 ± 9.47
	1 year	61.90 ± 8.03	48.93 ± 6.51	47.71 ± 7.08
AS-OCT (MGI)	6 months	112.45 ± 15.26	91.17 ± 13.49	80.64 ± 13.70
	1 year	93.82 ± 14.90	79.86 ± 13.19	77.66 ± 11.95

**Table 3:** Repeated-measures ANOVA results for BCVA, IVCM and AS-OCT analysis. ‘Time’ represents the main effect of time (comparison between 6- and 12-month visits), ‘Group’ represents the main effect of the surgical technique (VISCO, BSS, AIR), and ‘Time x Group’ their interaction. Significant main effects of both time and group were observed, while no significant interaction was detected, except for the AS-OCT analysis (*p* < 0.001)

		F	p	η²
BCVA	Time	77.619	< 0.001	0.581
	Group	3.864	0.027	0.121
	Time x Group	2.506	0.091	0.082
IVCM	Time	138.400	< 0.001	0.712
	Group	15.599	< 0.001	0.358
	Time x Group	1.633	0.205	0.055
AS-OCT	Time	184.634	< 0.001	0.767
	Group	16.858	< 0.001	0.376
	Time x Group	30.975	< 0.001	0.525

## Discussion

The choice of UT-DSAEK for this study was based on evidence from recent meta-analyses indicating that ultra-thin grafts (thinner than 100 μm [22, 23]) provide visual outcomes comparable to DMEK while retaining the procedural simplicity and lower complication rates of conventional DSAEK [6]. Although DMEK may offer slightly superior visual recovery in the early postoperative period, long-term outcomes tend to level off, with UT-DSAEK achieving similar final visual results [5]. Moreover, DMEK is associated with higher complication rates, particularly the need for rebubbling, which can adversely affect interface morphology, potentially influencing both adhesion and optical performance [4].

This highlights the critical importance of the graft–host interface, which represents the central determinant of endothelial keratoplasty success. Structural alterations at this level may manifest as increased interface reflectivity on imaging, a phenomenon that has been described in the literature within the spectrum of textural interface opacity (TIO) [7, 8, 11].

Even subtle alterations in its morphology can affect visual recovery and, in some cases, predispose to postoperative complications such as persistent haze, fibrosis or interface fluid retention, processes than may be influenced by intraoperative factors.

Detailed morphological analysis of this zone, therefore, provides valuable insight into the mechanisms of visual restoration and can guide optimization of surgical techniques and intraoperative maneuvers.

At histological level, the study by Weis et al. [24] in a DSAEK animal model suggested that increased interface reflectivity may result not from myofibroblast activation, but from keratocyte apoptosis and the formation of an acellular zone. These structural changes in the extracellular matrix and alterations in stromal cellularity may play a significant role in modulating light scatter and interface transparency, particularly in the early postoperative phase.

The role of transient interface fluid (TIF) in the development of postoperative interface haze has been emphasized in the PIONEER study [10, 25, 26], one of the first large-scale investigations to use intraoperative OCT (iOCT) to explore graft-host interface dynamics during DSAEK. Their findings demonstrated a strong association between the presence of TIF after lenticule positioning and the subsequent development of textural interface opacity (TIO), suggesting that early occult fluid pockets may precipitate structural changes leading to increased reflectivity. Similarly, Chatzea et al. [27] introduced the “M-TIO grading scale”, an OCT-based classification system designed to objectively screen and grade TIO in DSAEK grafts prior to transplantation. Both studies highlight the influence of intraoperative or even preoperative factors on the eventual optical quality of the interface. More recently, an additional study [28] has suggested that parameters related to graft preparation, particularly microkeratome dissection characteristics such as the relationship between pre-cut graft thickness, lenticule thickness, and microkeratome head size, may influence the risk of developing TIO. These findings further support the concept that preoperative graft morphology and preparation techniques may contribute to the structural characteristics of the postoperative interface.

However, while these studies have provided important insights into preoperative graft characteristics and intraoperative interface dynamics, standardized longitudinal in vivo postoperative evaluations of interface reflectivity remain limited.

Moreover, to our knowledge, no previous studies have quantitatively assessed how specific intraoperative surgical techniques may influence the postoperative expression of interface opacity. Therefore, a detailed and quantitative assessment of the interface is essential not only to clarify the mechanisms underlying visual outcomes but also to identify early structural changes that may precede clinically significant complications. Comparing quantitatively different Descemet stripping techniques using our MGI-based approach, we provide continuous, objective measurements that implement these earlier studies, allowing for a more detailed and reproducible assessment of microstructural organization and optical properties of the graft–host interface over multiple postoperative timepoints.

Building on this rationale, the present study provides a comprehensive morphological assessment of the graft-host interface following UT-DSAEK, comparing three different Descemet stripping techniques, performed with ophthalmic viscosurgical device, balanced salt solution, or air. Integrating IVCM and AS-OCT, we aimed to understand how intraoperative factors may be associated with the morphology and reflectivity of the interface.

### Functional outcomes

Although the primary focus of this study was the morphological characterization of the interface, BCVA was also evaluated to explore its correlation with structural findings. All groups showed significant visual improvement over time, confirming effective visual rehabilitation following UT-DSAEK. Notably, at 6 months, the AIR group showed faster BCVA recovery compared to VISCO and BSS groups, consistent with the lower interface reflectivity measured with both IVCM and AS-OCT and reinforcing the relationship between interface optical quality and postoperative visual performance [7, 8, 10, 19, 29]. Heinzelmann et al. [29] showed a strong inverse correlation between interface backscatter and BCVA using Scheimpflug imaging, indicating that higher light scatter at the graft-host interface negatively impacts visual recovery. Similar results were confirmed using IVCM, where elevated interface reflectivity and the presence of hyperreflective particles were linked to reduced visual quality [19]. By 12 months, visual outcomes were comparable across all groups, suggesting that progressive healing and stromal reorganization contribute to gradual improvement in interface transparency over time.

### In vivo confocal microscopy

IVCM enables microstructural evaluation of activated keratocytes, interface debris, and changes in stromal cellularity, all of which contribute to optical scatter. Quantitative IVCM analysis revealed that the VISCO group exhibited significantly higher interface reflectivity than both BSS and AIR groups at 6 and 12 months. Despite these higher absolute values, the magnitude of reflectivity reduction over time was comparable across groups, consistent with previous long-term confocal studies showing gradual reduction of interface haze following DSAEK [30]. This suggests that although the use of a viscoelastic substance initially produces a brighter interface, the progressive reduction in reflectivity indicates that remodeling processes may proceed even more actively at later stages, possibly reflecting accelerated removal of residual material and stromal reorganization. Conversely, BSS- and air-assisted techniques started from lower baseline values and showed a similar decline in reflectivity.

### Anterior segment – optical coherence tomography

AS‑OCT provides a non‑invasive assessment of the graft‑host interface and, unlike IVCM, is not capable of detecting microstructural features at cellular level (such as activated keratocytes, individual debris particles, or cellular‑matrix changes). Rather, AS‑OCT provides an overall assessment of reflectivity and light scattering across the entire graft‑host interface, evaluating large‑scale optical changes over time. In the present study, AS-OCT revealed persistently higher MGI in the VISCO group compared to the BSS and AIR groups at both 6 and 12 months. Interestingly, despite this initial difference, the rate of decline in reflectivity over the follow‑up period was slightly faster in the VISCO group, suggesting that the initial ‘cost’ of a brighter interface might be compensated by a more active subsequent remodeling phase, which may eventually lead to a leveling of interface reflectivity with the other groups in the long term. These findings are in line with previous reports [31–33] indicating that textural interface opacities may resolve spontaneously over the long term, progressively reducing their optical impact and clinical relevance. In contrast, the BSS and AIR groups started from lower baseline MGI values and showed a slightly slower rate of decline compared to VISCO, which may reflect a less pronounced initial deposition of residual material.

### Possible mechanisms and clinical implications

**Fig. 8:**
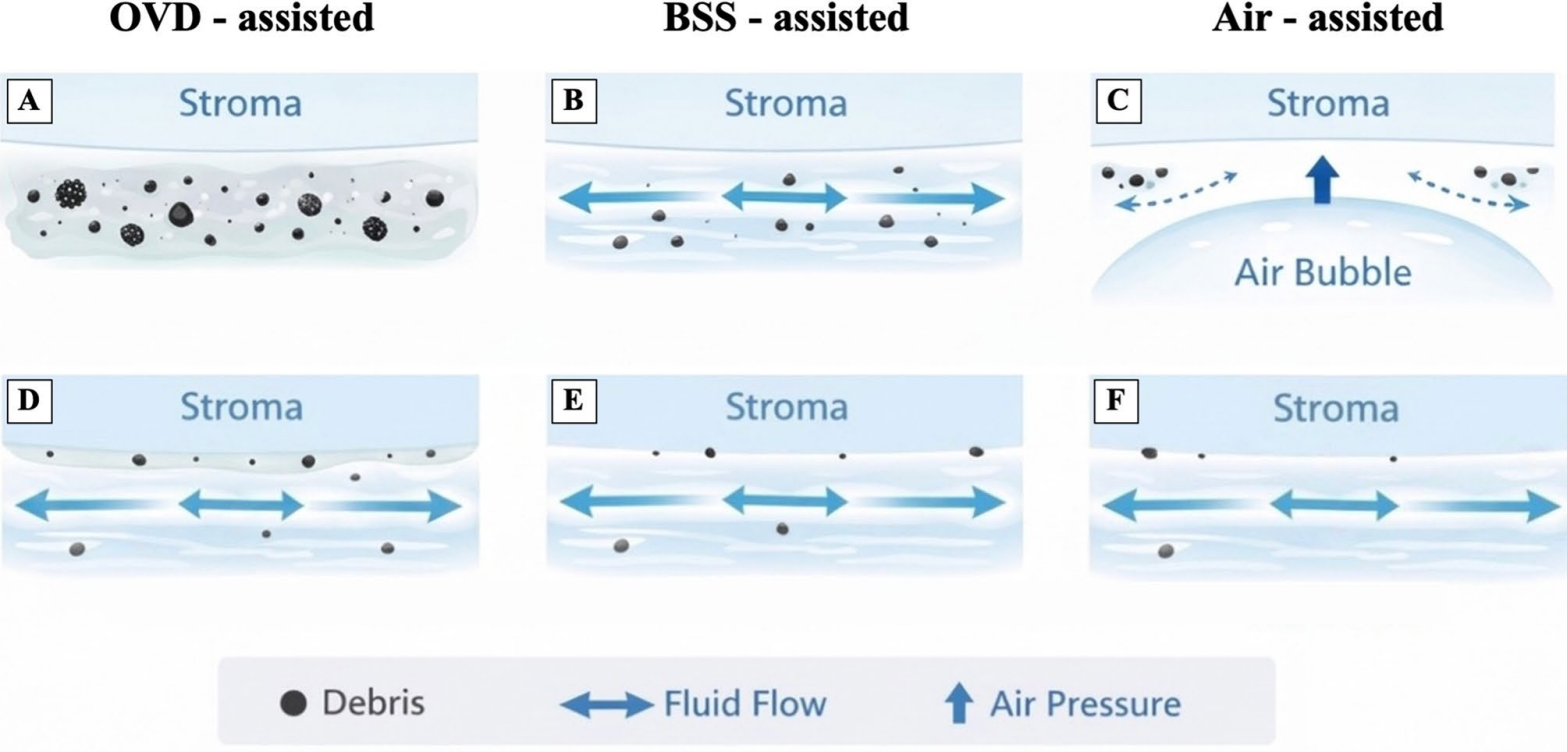
Schematic illustration of the potential mechanisms influencing graft–host interface reflectivity according to the Descemetorhexis technique. Panels **A**–**C **represent the Descemetorhexis phase performed under different intraoperative environments: OVD-assisted (**A**), BSS-assisted (**B**), and air-assisted (**C**). In the OVD-assisted technique (**A**), viscoelastic material may trap particulate debris within the interface, whereas continuous BSS flow (**B**) or air pressure (**C**) may facilitate the removal of debris from the stromal surface. Panels **D**–**F** illustrate the subsequent surgical phase before graft implantation, when the anterior chamber is typically maintained under BSS infusion in all techniques. Residual OVD (**D**) may remain adherent to the stromal surface despite OVD removal and BSS irrigation, while BSS (**E**) or air (**F**) allow more effective debris clearance, resulting in a cleaner interface prior to graft insertion

The persistently higher reflectivity observed in the VISCO group may be attributed to residual viscoelastic device trapped within the graft-host interface. In addition to creating a physical barrier that affects adhesion and light transmission, the viscoelastic material may change the composition and consistency of the extracellular matrix, resulting in a more reflective interface. Recent studies [11, 28] have suggested that TIO may arise from the interaction between retained viscoelastic material and irregular stromal fibers of the donor graft, which can promote the entrapment of particulate debris, delaying the physiological cleaning and remodeling processes that normally occur at the interface. This hypothesis is consistent with previous reports of interface haze and particulate debris following intraoperative OVD use [34]. Interface debris have been suggested to originate from the microkeratome instrumentation used to create the flap [13]. In addition, minor stromal manipulation of the recipient bed with surgical instruments during Descemet stripping, as well as particulate material freely circulating within the anterior chamber during surgery, may represent additional potential sources of interface debris that can subsequently become trapped at the graft–host junction. Although these particles may influence optical clarity, their clinical significance remains uncertain. In confocal microscopy studies of DSAEK patients, birefringent particles present at graft-host interface were not correlated with postoperative visual acuity, suggesting that the presence of interface debris does not necessarily predict visual outcomes [35, 36]. A schematic representation of these possible mechanisms is illustrated in Fig. 8 (A, D), which shows how a thin residual layer of OVD may persist on the stromal surface after Descemetorhexis and potentially trap debris despite subsequent OVD removal and BSS irrigation.

In contrast, performing Descemet stripping under air or BSS likely facilitates more complete removal of debris and improves graft–host apposition, potentially resulting in lower interface reflectivity and faster functional recovery [21]. As shown in Fig. 8 (B, C), continuous BSS flow or air pressure promotes effective removal of debris from the stromal surface, leading to a cleaner interface prior to graft insertion (E, F). Overall, these results provide clinical evidence that intraoperative environments favoring viscoelastic persistence may contribute to increased early postoperative interface reflectivity, consistent with the mechanisms proposed in recent studies on TIO [11, 28, 34], while techniques promoting debris clearance are associated with better early interface clarity. These findings underscore that intraoperative choices, even if their effects attenuate over time, play a relevant role in shaping early postoperative outcomes and should therefore be considered clinically meaningful.

Only a few studies in the literature have combined both IVCM and AS-OCT to obtain a comprehensive characterization of the graft–host interface. Among them, Mencucci et al. [37] provided one of the limited simultaneous assessments using both modalities, correlating structural findings with postoperative visual outcomes. Our study expands this limited evidence by adopting a standardized quantitative method based on MGI, improving measurement reproducibility and allowing direct comparison of reflectivity across imaging modalities and time points. The combined use of IVCM and AS-OCT allowed precise characterization of interface reflectivity and the detection of subtle temporal changes, reinforcing the concept that early interface optimization is a key determinant of postoperative visual rehabilitation, even in the presence of long-term convergence.

### Limitations

Nevertheless, several limitations should be acknowledged in the present study. First, the relatively small sample size and the single-surgeon design may limit the generalizability of results and introduce operator bias. In addition, the retrospective nature of the study and the lack of randomization in the allocation of Descemetorhexis techniques may have introduced selection bias, as surgical approach was based on surgeon preference and intraoperative considerations.

Moreover, although BCVA was included to provide a functional context for the morphological findings, it represents only a partial measure of visual quality. Parameters such as higher-order aberrations, contrast sensitivity, or patient-reported outcome measures (PROMs) were not assessed; these functional metrics may provide a more comprehensive evaluation of optical performance after endothelial keratoplasty. In addition, a formal statistical correlation analysis between interface reflectivity (MGI) and BCVA was not performed. Therefore, the present findings should be interpreted with caution in terms of structure–function relationships. Nevertheless, the observed trends may provide a useful basis for future studies specifically designed to integrate morphological and functional outcomes.

Future prospective randomized studies integrating multimodal imaging with advanced visual quality metrics, as well as combined preoperative M-TIO grading and postoperative in vivo interface reflectivity analysis, are warranted to better elucidate the clinical implications of graft–host interface morphology and the relationship with functional visual performance following UT-DSAEK.

## Conclusion

In conclusion, this study provides the first detailed, comparative morphological evaluation of the UT-DSAEK interface based on the Descemetorhexis technique, integrating IVCM and AS-OCT assessments. The combined use of these two instruments, along with a standardized quantitative analysis based on MGI, allowed for a reliable and detailed assessment of interface reflectivity over time.

Overall, viscosurgical device-assisted procedures are associated with higher interface reflectivity at both 6 and 12 months, as shown by both imaging modalities. However, the observed trend of progressive reflectivity reduction in the OVD-assisted group suggests that, over the long term, interface may eventually reach levels similar to those of the other groups. In contrast, BSS- and especially air-assisted procedures showed lower initial reflectivity and earlier functional recovery, aligning with the hypothesis that these media promote more complete intraoperative debris removal. From a clinical perspective, minimizing the use of viscoelastic substances during Descemetorhexis, when feasible, may be associated with improved early interface clarity and faster visual recovery, while their use may be more commonly justified in combined procedures, such as those associated with concomitant cataract surgery.

These findings highlight the impact of intraoperative choices on early interface reflectivity, their association with visual outcomes, and the ongoing remodeling processes that may ultimately normalize interface characteristics across different surgical approaches. This multimodal approach provides additional evidence supporting the refinement of surgical strategies to improve graft–host integration and visual rehabilitation.

## Data Availability

The datasets generated and/or analysed during the current study are not publicly available, but are available from the corresponding author on reasonable request.

## References

[1] Deng SX, Lee WB, Hammersmith KM, Kuo AN, Li JY, Shen JF, et al. Descemet Membrane Endothelial Keratoplasty: Safety and Outcomes: A Report by the American Academy of Ophthalmology. Ophthalmology. 2018;125:295–310. 28923499 10.1016/j.ophtha.2017.08.015

[2] Price FW, Price MO. Descemet’s stripping with endothelial keratoplasty in 200 eyes: Early challenges and techniques to enhance donor adherence. J Cataract Refract Surg. 2006;32:411–8. 16631048 10.1016/j.jcrs.2005.12.078

[3] Koenig SB, Covert DJ, Dupps WJ, Meisler DM. Visual acuity, refractive error, and endothelial cell density six months after Descemet stripping and automated endothelial keratoplasty (DSAEK). Cornea. 2007;26:670–4. 17592314 10.1097/ICO.0b013e3180544902

[4] Sela TC, Iflah M, Muhsen K, Zahavi A. Descemet membrane endothelial keratoplasty compared with ultrathin Descemet stripping automated endothelial keratoplasty: a meta-analysis. BMJ Open Ophthalmol. 2023;8. 10.1136/bmjophth-2023-001397 PMC10626808 37914389

[5] Weisenthal RW, Yin HY, Jarstad AR, Wang D, Verdier DD. Long-term Outcomes in Fellow Eyes Comparing DSAEK and DMEK for Treatment of Fuchs Corneal Dystrophy. Am J Ophthalmol. 2022;233:216–26. 34157279 10.1016/j.ajo.2021.06.013

[6] Hurley DJ, Murtagh P, Guerin M. Ultrathin Descemet Stripping Automated Endothelial Keratoplasty (UT-DSAEK) versus Descemet Membrane Endothelial Keratoplasty (DMEK)-a systematic review and meta-analysis. Eye (Lond). 2023;37:3026–32. 36934158 10.1038/s41433-023-02467-2 PMC10516931

[7] Kim K, Alder B, Vora GK, Carlson AN, Afshari NA, Kuo AN, et al. Textural interface opacity after Descemet-stripping automated endothelial keratoplasty. J Cataract Refract Surg. 2014;40:1514–20. 25135544 10.1016/j.jcrs.2013.12.020

[8] Vira S, Shih CY, Ragusa N, Sheyman A, Feder R, Weisenthal RW, et al. Textural interface opacity after descemet stripping automated endothelial keratoplasty: A report of 30 cases and possible etiology. Cornea. 2013;32. 10.1097/ICO.0b013e31826429d5 23132442

[9] Kymionis GD, Ide T, Yoo SH. Interface wavelike deposits after descemet stripping automated endothelial keratoplasty. Arch Ophthalmol. 2009;127:1389–90. 19822861 10.1001/archophthalmol.2009.249

[10] Juthani VV, Goshe JM, Srivastava SK, Ehlers JP. Association between transient interface fluid on intraoperative OCT and textural interface opacity after DSAEK surgery in the PIONEER study. Cornea. 2014;33:887–92. 25055146 10.1097/ICO.0000000000000209 PMC4197138

[11] Newman LR, Rosenwasser GOD, Dubovy SR, Matthews JL. Clinicopathologic correlation of textural interface opacities in descemet stripping automated endothelial keratoplasty: A case study. Cornea. 2014;33:306–9. 24457450 10.1097/ICO.0000000000000057

[12] Moura-Coelho N, Papa-Vettorazzi R, Reyes A, Cunha JP, Güell JL. Ultrathin DSAEK versus DMEK - Review of systematic reviews. Eur J Ophthalmol. 2024;34:913–23. 37964555 10.1177/11206721231214605

[13] Kymionis GD, Kankariya VP, Kontadakis GA. Long-term presence of metallic particles in the DSAEK interface. Eye. 2011;25:1382. 21738228 10.1038/eye.2011.165 PMC3194318

[14] Shulman J, Kropinak M, Ritterband DC, Perry HD, Seedor JA, McCormick SA, et al. Failed descemet-stripping automated endothelial keratoplasty grafts: a clinicopathologic analysis. Am J Ophthalmol. 2009;148. 10.1016/j.ajo.2009.06.023 19674726

[15] Yu AC, Myerscough J, Socea S, Furiosi L, Spena R, Bovone C, et al. Interface Drainage and Antimicrobial Irrigation Avoid Repeat Keratoplasty for Post-DSAEK Cold Interface Abscess. Cornea. 2021;40:1207–10. 33782265 10.1097/ICO.0000000000002710

[16] Semeraro F, Di Salvatore A, Bova A, Forbice E. Etiopathogenesis and therapy of epithelial ingrowth after Descemet’s stripping automated endothelial keratoplasty. Biomed Res Int. 2014;2014. 10.1155/2014/906087 PMC4165875 25254218

[17] Letko E, Price DA, Lindoso EMS, Price MO, Price FW. Secondary graft failure and repeat endothelial keratoplasty after Descemet’s stripping automated endothelial keratoplasty. Ophthalmology. 2011;118:310–4. 20869118 10.1016/j.ophtha.2010.06.032

[18] Gadhvi K, Pagano L, Menassa N, Borroni D, Kaye SB, Levis HJ, et al. DSAEK Centration and Interface Folds: Surgical Management. Cornea. 2020;39:1457–9. 32618854 10.1097/ICO.0000000000002411

[19] Ferrari G, Reichegger V, Ludergnani L, Delfini E, MacAluso C. In vivo evaluation of DSAEK interface with scanning-laser confocal microscopy. BMC Ophthalmol. 2012;12. 10.1186/1471-2415-12-32 PMC3441224 22853313

[20] Khakshour H, Nikandish M, Salehi M, Ghooshkhanehei H, Vejdani A. Evaluation of interface reflectivity and corneal aberrations following Descemet’s stripping automated endothelial keratoplasty. Oman J Ophthalmol. 2019;12:108–13. 31198297 10.4103/ojo.OJO_188_2017 PMC6561048

[21] Gabbay IE, Bahar I, Nahum Y, Livny E. Comparison of Descemet stripping under continuous air flow, manual air injection and balanced salt solution for DMEK: a pilot study. Graefe’s Archive Clin Experimental Ophthalmol. 2017;255:1605–11. 10.1007/s00417-017-3675-0 28456826

[22] Dickman MM, Kruit PJ, Remeijer L, van Rooij J, Van der Lelij A, Wijdh RHJ, et al. A Randomized Multicenter Clinical Trial of Ultrathin Descemet Stripping Automated Endothelial Keratoplasty (DSAEK) versus DSAEK. Ophthalmology. 2016;123:2276–84. 27659544 10.1016/j.ophtha.2016.07.036

[23] Droutsas K, Petrelli M, Miltsakakis D, Andreanos K, Karagianni A, Lazaridis A, et al. Visual Outcomes of Ultrathin-Descemet Stripping Endothelial Keratoplasty versus Descemet Stripping Endothelial Keratoplasty. J Ophthalmol. 2018;2018:5924058. 30515318 10.1155/2018/5924058 PMC6237014

[24] Weis AJ, Huxlin KR, Callan CL, DeMagistris MA, Hindman HB. Keratocyte apoptosis and not myofibroblast differentiation mark the graft/host interface at early time-points post-DSAEK in a cat model. PLoS ONE. 2013;8. 10.1371/journal.pone.0075623 PMC3787047 24098706

[25] Hallahan KM, Cost B, Goshe JM, Dupps WJ, Srivastava SK, Ehlers JP. Intraoperative Interface Fluid Dynamics and Clinical Outcomes for Intraoperative Optical Coherence Tomography–Assisted Descemet Stripping Automated Endothelial Keratoplasty From the PIONEER Study. Am J Ophthalmol. 2017;173:16–22. 27702622 10.1016/j.ajo.2016.09.028 PMC5136509

[26] Ehlers JP, Dupps WJ, Kaiser PK, Goshe J, Singh RP, Petkovsek D, et al. The prospective intraoperative and perioperative ophthalmic imaging with optical CoherEncE TomogRaphy (PIONEER) study: 2-year results. Am J Ophthalmol. 2014;158:999–e10071. 25077834 10.1016/j.ajo.2014.07.034 PMC4250395

[27] Chatzea MS, Kymionis GD, Vakalopoulos DG, O’Brien RC, Mora D, Llanes K, et al. Screening and grading of textural interface opacities in DSAEK grafts with the M-TIO scale for predicting visual outcomes. Diagnostics. 2025;15. 10.3390/diagnostics15101241 PMC12109586 40428234

[28] Chatzea MS, Kymionis GD, Vakalopoulos DG, O’Brien RC, Mora D, Llanes K, et al. Impact of Microkeratome Dissection Parameters on Textural Interface Opacities in DSAEK Grafts. Diagnostics. 2025;15:1608. 40647607 10.3390/diagnostics15131608 PMC12249330

[29] Heinzelmann S, Böhringer D, Maier PC, Reinhard T. Correlation between visual acuity and interface reflectivity measured by pentacam following DSAEK. Acta Ophthalmol. 2014;92. 10.1111/aos.12217 23889769

[30] Baratz KH, McLaren JW, Maguire LJ, Patel SV. Corneal Haze Determined by Confocal Microscopy 2 Years After Descemet Stripping With Endothelial Keratoplasty for Fuchs Corneal Dystrophy. Arch Ophthalmol. 2012;130:868–74. 22410629 10.1001/archophthalmol.2012.73

[31] Kontadakis GA, Palioura S, Yoo SH. Wavelike Interface Opacities after Descemet-Stripping Automated Endothelial Keratoplasty: 7-Year Follow-up. Eye Contact Lens. 2017;43:e13–5. 26398577 10.1097/ICL.0000000000000195

[32] Shih CY, Ritterband DC, Rubino S, Palmiero PM, Jangi A, Liebmann J, et al. Visually Significant and Nonsignificant Complications Arising From Descemet Stripping Automated Endothelial Keratoplasty. Am J Ophthalmol. 2009;148:837–43. 19800608 10.1016/j.ajo.2009.06.034

[33] Patel SV, McLaren JW. In vivo confocal microscopy of fuchs endothelial dystrophy before and after endothelial keratoplasty. JAMA Ophthalmol. 2013;131:611–8. 23471204 10.1001/jamaophthalmol.2013.799

[34] Kymionis G, Voulgari N, Kontadakis G, Mikropoulos D, Petrovic A, Droutsas K. Surgical management of post-Descemet stripping automated endothelial keratoplasty interface haze associated with interface deposits. Indian J Ophthalmol. 2019;68:174. 10.4103/ijo.IJO_883_19 PMC6951205 31856502

[35] Espana EM, Huang B. Confocal microscopy study of donor-recipient interface after Descemet’s stripping with endothelial keratoplasty. Br J Ophthalmol. 2010;94:903–8. 19825836 10.1136/bjo.2009.165712

[36] Kobayashi A, Mawatari Y, Yokogawa H, Sugiyama K. In Vivo Laser Confocal Microscopy After Descemet Stripping with Automated Endothelial Keratoplasty. Am J Ophthalmol. 2008;145:977–e9851. 18400202 10.1016/j.ajo.2008.02.009

[37] Mencucci R, Favuzza E, Tartaro R, Busin M, Virgili G. Descemet stripping automated endothelial keratoplasty in Fuchs’ corneal endothelial dystrophy: Anterior segment optical coherence tomography and in vivo confocal microscopy analysis. BMC Ophthalmol. 2015;15. 10.1186/s12886-015-0096-x PMC4545854 26253099

